# Pediatric Neuromyelitis Optica Spectrum Disorder: Case Series and Literature Review

**DOI:** 10.3390/life12010019

**Published:** 2021-12-23

**Authors:** Michela Ada Noris Ferilli, Roberto Paparella, Ilaria Morandini, Laura Papetti, Lorenzo Figà Talamanca, Claudia Ruscitto, Fabiana Ursitti, Romina Moavero, Giorgia Sforza, Samuela Tarantino, Martina Proietti Checchi, Federico Vigevano, Massimiliano Valeriani

**Affiliations:** 1 Neuroscience Department, Bambino Gesù Children’s Hospital IRCCS, 00165 Rome, Italy; laura.papetti@opbg.net (L.P.); fabiana.ursitti@opbg.net (F.U.); romina.moavero@opbg.net (R.M.); giorgia.sforza@opbg.net (G.S.); federico.vigevano@opbg.net (F.V.); massimiliano.valeriani@opbg.net (M.V.); 2 Department of Maternal and Child Health and Urology, Sapienza University of Rome, 00165 Rome, Italy; roberto.paparella@uniroma1.it; 3 Child Neurology Unit, Systems Medicine Department, Tor Vergata University Hospital of Rome, 00165 Rome, Italy; ilaria.morandini@ptvonline.it (I.M.); claudia.ruscitto@ptvonline.it (C.R.); 4 Imaging Department, Bambino Gesù Children’s Hospital IRCCS, 00165 Rome, Italy; lorenzo.figatalamanca@opbg.net; 5 Unit of Clinical Psychology, Neuroscience Department, Bambino Gesù Children’s Hospital IRCCS, 00165 Rome, Italy; samuela.tarantino@opbg.net (S.T.); martina.proietti@opbg.net (M.P.C.); 6 Center for Sensory-Motor Interaction, Denmark Neurology Unit, Aalborg University, 9220 Aalborg, Denmark

**Keywords:** neuromyelitis optica spectrum disorder, aquaporin-4 antibody, myelin oligodendrocyte glycoprotein antibodies, optic neuritis, longitudinally extensive transverse myelitis, children

## Abstract

Neuromyelitis Optica Spectrum Disorder (NMOSD) is a central nervous system (CNS) inflammatory demyelinating disease characterized by recurrent inflammatory events that primarily involve optic nerves and the spinal cord, but also affect other regions of the CNS, including hypothalamus, area postrema and periaqueductal gray matter. The aquaporin-4 antibody (AQP4-IgG) is specific for NMOSD. Recently, myelin oligodendrocyte glycoprotein antibodies (MOG-IgG) have been found in a group of AQP4-IgG negative patients. NMOSD is rare among children and adolescents, but early diagnosis is important to start adequate therapy. In this report, we present cases of seven pediatric patients with NMOSD and we review the clinical and neuroimaging characteristics, diagnosis, and treatment of NMOSD in children.

## 1. Introduction

Neuromyelitis Optica Spectrum Disorder (NMOSD) is a rare central nervous system (CNS) inflammatory demyelinating disease characterized by recurrent inflammatory events, primarily involving optic nerves and the spinal cord, but also affecting other regions of the CNS, including hypothalamus, area postrema and periaqueductal gray matter.

It is stratified by the aquaporin-4 antibody (AQPA-IgG) into AQP4-IgG-positive NMOSD and seronegative NMOSD. AQP4-IgG is a sensitive and specific marker of NMOSD and has a function in its immunopathogenesis.

AQP4 is a water channel protein expressed on astrocyte foot processes at the blood-brain barrier, especially in the periaqueductal and periventricular regions and at the level of the spinal cord gray matter [1].

AQP4-IgG positive NMOSD could be classified as an autoimmune astrocytopathic disease, in which the AQP4-IgG is thought to cause the internalization and degradation of AQP4, dysregulation of water and glutamate homeostasis, activation of the classical complement pathway, and antibody-mediated cytotoxicity.

Recent studies have shown that neutrophils, macrophages, and natural killer (NK) cells contribute to the formation of lesions in NMOSD through different mechanisms, such as the production of elastase, the secretion of cytokines, phagocytosis, and antibody-dependent cytotoxicity [2]. Eosinophils are also involved in the pathogenesis of lesions in NMOSD, and these cells have been shown to contribute to tissue damage [3].

AQP4-IgG seronegativity in 10–25% of NMOSD patients suggests that there are other mechanisms involved in NMOSD pathogenesis [4]. More recently, myelin oligodendrocyte glycoprotein antibodies (MOG-IgG) have been found in a group of AQP4-IgG negative patients, which have a different pathogenesis, younger age at presentation, fewer relapses, and a better outcome [5,6,7].

Approximately 3–5% of NMOSD cases are reported to be pediatric onset, before 18 years of age [8,9,10,11]. Early differentiation of NMOSD from other acquired demyelinating syndromes (ADS) of the CNS, such as acute disseminated encephalomyelitis (ADEM) and multiple sclerosis (MS), is critical for starting opportune treatment. In fact, it is now known that some disease-modifying therapies used for MS, including interferons, natalizumab and fingolimod, are ineffective in patients with NMOSD and may precipitate the course of the disease [12,13,14].

In this article we present cases of seven pediatric patients with NMOSD and we review the literature related to clinical and neuroimaging characteristics, diagnosis and treatment of pediatric NMOSD.

## 2. Clinical Cases

Clinical, radiological, and laboratory findings of our patients are reported in Table 1.

### 2.1. NMOSDs with AQP4-IgG

#### 2.1.1. Case 1

**Figure 1:**
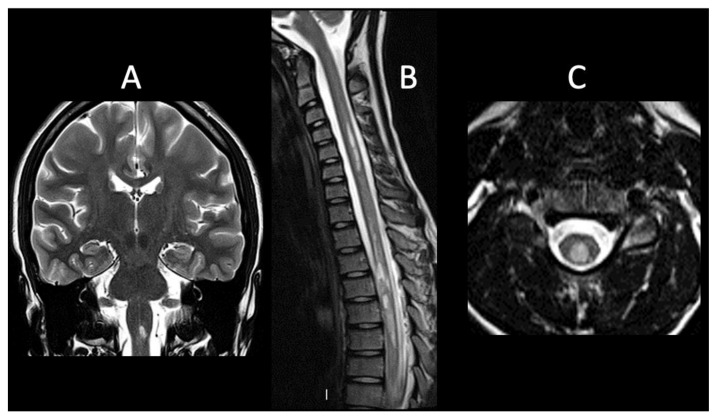
NMOSDs with AQP4-IgG. (**A**). Coronal T2-weighted image (WI) shows hyperintense lesion in brainstem (case 1), (**B**). sagittal T2-WI shows multiple and confluent hyperintense lesions in cervical and dorsal spine consistent with LETM (case 2), (**C**). axial T2-WI shows hyperintense lesion in cervical spine (case 2).

Patient 1 is a 16-year-old female who reported headache, vomiting and vertigo. Magnetic resonance imaging (MRI) showed multiple T2-hyperintense lesions with gadolinium enhancement in the brain, brainstem, and dorsal spine (Figure 1A). Cerebrospinal fluid (CSF) studies showed an increased number of lymphocytes, in absence of oligoclonal bands (OCBs). Expanded Disability Status Score (EDSS) was 3 at this time. Diagnosis of NMOSD was confirmed by the positivity of AQP4-IgG [15]. Given the positivity of anti–Sjögren’s-syndrome-related antigen A autoantibodies (anti-SSA/Ro), the presence of eye dryness, and the evidence of lung interstitiopathy at chest computed tomography (CT) and spirometry, a further diagnosis of Sjögren’s syndrome was made. She has been treated with high-dose steroids, hydroxychloroquine, mycophenolate mofetil (MMF), and rituximab (RTX). One year after diagnosis, AQP4-IgG became negative. At the time of last follow-up, 41 months after diagnosis, MRI showed stability of preexisting lesions, with no gadolinium enhancement, and in absence of new lesions. EDSS was 0. She was daily taking prednisone, hydroxychloroquine, and MMF. AQP4-IgG remained negative.

#### 2.1.2. Case 2

Patient 2 is a female who presented at the age of 9 years with bilateral optic neuritis (ON) and progressive hyposthenia of the lower limbs. MRI investigation revealed multiple T2-hyperintense lesions in the right frontal subcortical region, corpus callosum, and in the cervical and dorsal spine (Figure 1B,C). Diagnosis of NMOSD was then confirmed by the positivity of AQP4-IgG [15]. CSF OCBs were present. EDSS was four at the time of the first attack. Hematopoietic stem cell transplantation (HSCT) from the HLA-haploidentical father was performed, after several relapses due to an ineffective disease control (EDSS was 6.5) despite treatment with high-dose steroids, azathioprine (AZA), cyclosporine, and RTX. At the time of last follow-up, 42 months after HSCT, MRI showed a significant reduction of preexisting lesions with no gadolinium enhancement in absence of new lesions. The patient had no re-exacerbation, showing improvement in EDSS which became 5. AQP4-IgG remained positive.

#### 2.1.3. Case 3

Patient 3 is an 8-year-old female who presented with bilateral ON (EDSS was 4). MRI showed T2-hyperintensity of both optic nerves, with gadolinium enhancement on the left side. Visual evoked potentials (VEPs) showed a bilateral increase in latency. CSF OCBs were negative. Diagnosis of NMOSD was then confirmed by the positivity of AQP4-IgG [15]. After initial steroid treatment, she underwent plasmapheresis, followed by RTX. At the last follow-up, 19 months after diagnosis, MRI showed a significant reduction of preexisting lesions, with the patient undergoing the third cycle of RTX in absence of re-exacerbations. EDSS was 0.

### 2.2. NMOSDs without AQP4-IgG

#### 2.2.1. Case 4

**Figure 2:**
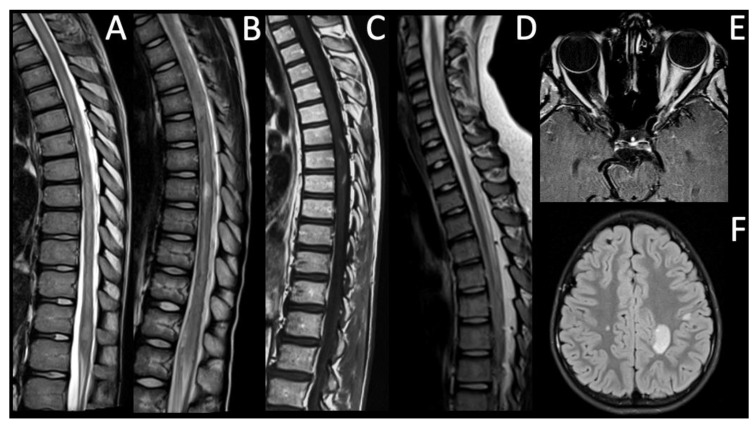
NMOSDs without AQP4-IgG. (**A**). Sagittal T2-WI shows multiple and confluent hyperintense lesions in the cervical and dorsal spine consistent with LETM (case 4), (**B**). sagittal T2-WI shows multiple and confluent hyperintense lesions in the dorsal spine extending from level T5 to the conus medullaris (case 5), (**C**). sagittal T1-WI acquired after the intravenous (iv) administration of gadolinium (gd) shows intramedullary enhancement between T6 and T8 levels (case 6), (**D**). sagittal T2-WI shows hyperintense lesions in the cervical spine (C2–C7) consistent with LTM (case 7), (**E**). axial T1-WI acquired after iv administration of gd shows left optic nerve enhancement consistent with left ON (case 7), (**F**) axial T2-FLAIR shows multiple brain hyperintense lesions (case 5).

Patient 4 is a male affected by ulcerative colitis since he was 4. He underwent a subtotal colectomy at the age of 12, because of a poor disease control. At the age of 15, he presented with a progressive right lower limb paresis; MRI detected multiple T2-hyperintense lesions extending from the cervical spine to the conus medullaris, with no gadolinium enhancement (Figure 2A). Serum AQP4-IgG, MOG-IgG and CSF OCBs were negative. VEPs showed a bilateral increase in latency, in the absence of visual symptoms. Motor evoked potentials (MEPs) and somatosensory evoked potentials (SEPs) were abnormal. EDSS at the time of first attack was 8.5. He presented acute myelitis with longitudinally extensive transverse myelitis (LETM) and increased latency to VEPs, so a suspected NMOSD diagnosis was made [15]. After an initial response to steroids, six months later, he had a clinical and radiological relapse with a new cerebral T2-hyperintense lesion in the cerebral deep white matter and gadolinium-enhancement of previous spinal lesions. In particular, the inflammatory brain lesion was not typical for NMOSD [15]. Therefore, RTX was administered. Maintenance therapy with MMF was started and then discontinued at age 19 due to clinical stability. At the time of last follow-up, 60 months after diagnosis, MRI and electrophysiologic studies were normal, with an EDSS of 0.

#### 2.2.2. Case 5

Patient 5 is a 10-year-old male who presented with a right lower limb paresis; sensory level was T10–T11. MRI showed multiple T2-hyperintense lesions with gadolinium enhancement in both cerebral hemispheres and in the cervical spine (Figure 2B,F). EDSS was 9 at diagnosis. VEPs showed a bilateral increase in latency, especially on the left side. MEPs and SEPs indicated an impairment of motor and somatosensory pathways of lower limbs. Serum AQP4-IgG, MOG-IgG and CSF OCBs were negative. Serum and CSF serology for neurotropic pathogens was negative. After the initial treatment with high-dose steroids, he developed paraplegia and left upper limb paresis, urinary and fecal incontinence, with a sensory level at T5–T6. T2-hyperintense lesions had extended towards conus medullaris. Suspected seronegative NMOSD diagnosis was made [15].

Therefore, he underwent PLEX followed by RTX, with a partial clinical improvement. At the time of last follow-up, 63 months after diagnosis, he presented with paraplegia, scoliosis, neurogenic bladder, and bowel dysfunction. EDSS was 7.5.

#### 2.2.3. Case 6

Patient 6 is a 13-year-old male who presented with gait ataxia, bilateral lower limb paresthesia, and pyramidal signs. Sensory level was T10. MRI showed hyperintense intramedullary signal at T2-weighted imaging between T5 and T9 (Figure 2C), with gadolinium enhancement. The patient did not report visual disturbances but had a concomitant bilateral increase in latency of VEPs that led to a suspected NMOSD diagnosis [15]; AQP4-IgG and MOG-IgG have always been negative. EDSS was 6 at onset. CSF OCBs were negative. He has shown a good response to steroids, initially administered intra-venously and subsequently orally as chronic therapy. At the last follow-up, 24 months after diagnosis, MRI showed stability of preexisting spinal lesions. EDSS was 0. Left eye function was recovered, while an increased latency of right VEPs persisted.

#### 2.2.4. Case 7

Patient 7 is a 11-year-old male who presented with left ON. MRI showed T2-hyperintense lesions with gadolinium enhancement in left optic nerve (Figure 2E) and T2-hyperintense medullary lesions with gadolinium enhancement between C2 and C7 levels consistent with LETM (Figure 2D). The patient had no signs or symptoms related to spinal cord lesion. Serum AQP4-IgG, MOG-IgG and CSF OCBs were negative, but an increased number of lymphocytes were found at CSF analysis.

MEPs and SEPs were normal and VEPs showed an increased latency only on the left side. EDSS was 2 at onset. Seronegative NMOSD diagnosis was made [15]. He has been treated with high-dose steroids intravenously, and then with oral steroids but, approximately five months later, the patient presented with right ON. Maintenance therapy was started with 7 cycles of intravenous immunoglobulin (IVIG). At the last follow-up, 24 months after diagnosis, he presented clinical and radiological stability, EDSS was 0.

## 3. Literature Review

### 3.1. Methods

This is a narrative review in which we have identified studies describing cases of NMOSD in pediatric age. 

The research was performed in the PUBMED database (last search was performed on 22th December 2021) with no limited period range. The following search terms were used to track articles: neuromyelitis optica and children. 

Our review included original studies and case reports exclusively on pediatric patients with NMOSD. 

We excluded all reviews and studies in which the sample included adult patients or pediatric and adult patients with NMOSD or those with other ADS of the CNS, such as ADEM, MS and MOG associated diseases.

### 3.2. Results

After the initial identification of 757 papers, we selected for the final analysis 41 manuscripts fulfilling our inclusion criteria. Table 2 shows the main characteristics of all studies selected for this review. 

## 4. Discussion

### 4.1. Epidemiology

Pediatric patients with NMOSD are older than children with ADEM but about the same age as those with MS [10,18]. Moreover, they have a worse prognosis than those affected by MS, as reported by Tillema et al. [55]. Further, Yara Dadalti Fragoso et al. showed that morbidity in patients with early onset NMOSD was higher than in those with early onset MS, reporting that 40% of their patients had achieved an EDSS of 7 or more within 6 years [16]. According to most reports NMOSD is more common in females than men [10,18], and the prevalence varies by ethnicity, being greater in non-Caucasian than in Caucasian populations [56].

In a large study conducted at the Mayo Clinic [10] in pediatric patients with NMOSD, median age at symptom onset was 12 years (range 4–18), there was a clear female pre-ponderance (88% were girls) and mixed ethnic background.

In another US pediatric population study [18], mean patient age at symptom onset was 10.2 ± 4.7 years. In patients <11 years of age female/male ratio was 1.5/1 while in those >11 years was 3.25/1. As for ethnicity, 37% were African American, 11% were Asian and 13% of patients had Hispanic/Latino ethnicity.

In a Brazilian study of children with NMOSD the median age at onset was 13 years (range 5–17), female/male ratio was 2.6/1, and the preponderance of patients were of mixed ethnicity (Caucasian and African) [16].

In our series, the median age of presentation was 12 years (range 8–16), all AQP4-IgG positive patients were female and all seronegative NMOSD patients were male. Six patients were Caucasian, and one was non-Caucasian.

### 4.2. Diagnostic Criteria

For many years, Neuromyelitis Optica (NMO), previously called Devic’s disease, has been identified as a subtype of MS. In 2004, with the discovery of a disease specific NMO-IgG antibody against the AQP4 water channel (AQP4-IgG), NMO was considered as a different autoimmune disease entity [1,57].

In 2006, Wingerchuk and colleagues [58] proposed the diagnostic criteria for NMO that require ON, transverse myelitis (TM), and at least two of three supportive criteria: contiguous spinal cord MRI lesion extending over three or more segments, brain MRI non-diagnostic for MS, or AQP4-IgG seropositivity. In 2015, new diagnostic criteria were created using nomenclature that defines the term NMOSD and stratifies patients by serologic testing in NMOSD with and without AQP4-IgG [15].

According to these criteria, six core clinical characteristics for diagnosis of NMOSD were identified, including ON, TM, area postrema syndrome, acute brainstem syndrome, symptomatic narcolepsy or acute diencephalic clinical syndrome, and symptomatic cerebral syndrome. For diagnosis in AQP4-IgG positive NMOSD patients, only one core criterion is enough. For seronegative NMOSD patients, two core criteria are necessary for diagnosis occur as a consequence of one or more clinical events, and at least one of these two core criteria should be ON, TM with longitudinally extensive transverse myelitis (LETM), or area postrema syndrome. Moreover, in seronegative NMOSD patients are necessary, dissemination in space (two or more different core clinical characteristics) with additional MRI requirements specific for each clinical syndrome.

These diagnostic criteria have been validated in the pediatric group [18].

### 4.3. Clinical Features

The most frequent presenting features of NMOSD are visual, motor, sensory and constitutional symptoms (such as vomiting, fever, and seizures). In most pediatric reports of NMOSD, ON occurred as the first clinical event in 50–75% of patients and TM in 30–50%, either alone or in combination [11]. Other symptoms are represented by ataxia, encephalopathy, and cranial nerve dysfunction, such as ophthalmoparesis or area postrema syndrome [59].

ON is defined as inflammation of the optic nerve and typically presents as loss of visual acuity accompanied by pain with eye movements and may be unilateral or bilateral. ON in NMOSD is commonly bilateral, longitudinally extensive, with a predilection for posterior optic nerve segments, particularly the optic chiasm [54,60].

Another typical clinical feature is TM, defined as a spinal cord inflammation causing sensory, motor, and autonomic disorders. LETM consists of contiguous inflammatory lesions extending over three or more vertebral segments [61]. The clinical presentation of a patient with TM or LETM consists of para or tetraplegia, depending on the spinal cord level involved and with a sensory and sphincter dysfunction [62]. In pediatric patients, LETM is less specific for NMOSD and may be present in children with MS or ADEM [63].

Area postrema syndrome is one of the core clinical characteristics for NMOSD, due to the involvement of this circumventricular organ located in the caudal part of the fourth ventricle [25,27,64]. It presents with intractable vomiting, nausea, and hiccups secondary to inflammation in the emetic reflex center situated in this area [62].

Another core criterion is acute brainstem syndrome reported in 40% of pediatric NMOSD patients [62]. The involvement of this region causes a dysfunction of cranial nerves (facial palsy, trigeminal autonomic cephalalgia). In most cases, an involvement of the oculomotor nerves which presents with diplopia or nystagmus can be observed [63].

Other clinical events are dysarthria, vestibular ataxia, or the involvement of the respiratory center [65].

The involvement of thalamus/hypothalamus could be observed in these patients and could cause acute diencephalic clinical syndrome. Hypotension, hypersomnia, behavioral changes, amenorrhea galactorrhea syndrome and narcolepsy are manifestations that can occur with the diencephalic involvement [62]. Syndrome of inappropriate antidiuretic hormone secretion (SIADH) is a common clinical presentation, characterized by excessive release of antidiuretic hormone (ADH) causing hyponatremia and is responsible for symptoms such as nausea, vomiting, irritability and in severe cases, seizures, and coma [66]. There are no data on the incidence of these symptoms in the pediatric population. 

Other rare neurological presentations in NMOSD are represented by cerebral syndromes that are reported in 16–32% of AQP4-IgG positive children [8]. This ADEM-like phenotype has been reported in pediatric patients positive for MOG-IgG or AQP4-IgG [67]. A large cerebral hemispheric lesion could cause hemiparesis, visual field involvement, and signs of encephalopathy [8].

About 40% of pediatric patients with NMOSD have comorbid autoimmune diseases [54], including organ-specific disorders (e.g., myasthenia gravis, thyroid disease, ulcerative colitis, celiac disease, primary sclerosing cholangitis and idiopathic thrombocytopenic purpura) and non-organ-specific disorders (e.g., systemic lupus erythematosus, Sjögren syndrome and antiphospholipid syndrome) [27,68].

One patient of ours with AQP4-IgG positive NMOSD (case 1) also had a diagnosis of Sjögren Syndrome. There are different case series that reported the association between pediatric NMOSD and Sjögren Syndrome [20,69].

According to recent studies, NMOSD can coexist with anti-N-methyl-D-aspartate receptor (NMDAR) encephalitis [70]. Anti-NMDAR encephalitis should be considered when NMOSD patients show atypical symptoms (abnormal behavior, psychiatric manifestations, autonomic dysfunction), or atypical brain lesions [71].

### 4.4. Imaging Findings

MRI is important in the diagnosis and follow-up of patients with NMOSD. ON is typically characterized as bilateral and longitudinally extensive over 1/2 optic nerve length, frequently affecting posterior optic pathway, particularly the optic chiasm [72].

During the acute and subacute phases, optic nerve MRI shows hyperintensity in T2-weighted sequences and gadolinium enhancement in T1 weighted-images, while optic nerve atrophy and variable hyperintensity on T2-weighted images are observed in chronic stages [73]. In NMOSD pediatric patients with ON can be observed infraorbital fat gadolinium enhancement [11].

LETM is highly suggestive of NMOSD, usually characterized by extensive centrally located hyperintensity in T2 weighted images extending over three or more vertebral segments and involving both white and gray matter [74]. Cervical and thoracic spinal segments are typically compromised [74]. In the acute phase LETM is characterized by cord swelling and gadolinium enhancement in T1-weighted sequences, while in the chronic phase the spinal cord may appear atrophic [75]. In children, LETM is less specific for NMOSD than in adults and may be observed also in patients with MS, ADEM and monophasic TM [60,76,77].

Brain lesions are typically localized in areas of high AQP4 expression, such as circumventricular organs around the third and fourth ventricles (diencephalon and brainstem), including hypothalamic, thalamic and the periaqueductal area, supratentorial and infratentorial white matter, midbrain, ependymal surfaces of the cerebellum and corpus callosum [78]. Lesions in the corpus collosum are often large and follow the ependymal line with various shapes, different from those typical of MS, that are small and ovoid lesions [78]. The involvement of area postrema is one of the most typical brain features in patients with NMOSD [78].

During acute phases, brain lesions show hyperintensity on FLAIR/T2-weighted images and variable enhancement on T1-weighted images [79]. Gadolinium enhancement may be seen in approximately 30% of pediatric patients with brain lesions, usually with a cloud-like pattern of enhancement, as in adults [8,10]. Although the brain areas involved are the same in adult and pediatric patients, lesions are usually more common and larger (>2 cm) in children than in adults [8,10].

### 4.5. Laboratory Features

The AQP4-IgG is a specific serum marker for NMOSD [42], described for the first time in 2004 by Lennon et al. [58]. The International Panel for NMO Diagnosis (IPND) recommended testing with a cell-based serum assay which has estimated sensitivity and specificity of 76.7% and 99.8%, respectively [15]. Testing for AQP4-IgG should be performed on serum, as it is more sensitive than CSF analysis. Data indicate significantly higher serum AQP4-IgG titer than CSF, as AQP4-IgG is produced in peripheral lymphoid tissues rather than intrathecally [80]. In a US study of pediatric NMOSD 65% of children were positive for AQP4-IgG and some patients became seropositive more than 3 years after onset [18]. Similar to adults, seropositivity for AQP4-IgG in children is usually associated with a relapsing course [76]. It is recommended to provide the dosage during the attack and before immunotherapy, because it may cause conversion to the negative state [80,81].

Children with NMOSD should also be tested for MOG-IgG. MOG-IgG has been found in a subgroup of adult and pediatric seronegative NMOSD patients [26]. Several studies have demonstrated that NMOSD patients with MOG-IgG usually have a younger age at presentation, fewer relapses, better outcomes, and are less frequently female than AQP4-IgG positive patients [7,82]. MOG-IgG have also been associated with other ADS, including ADEM, monophasic or recurrent ON, ON following an ADEM onset (ADEM-ON) and TM [7]. The development of a sensitive and specific test for MOG-IgG has permitted the assessment of the clinical and radiological characteristics of patients [83,84]. Our case series did not include NMOSD patients with MOG-IgG.

CSF OCBs, a hallmark of MS, are absent in most NMOSD patients. According to some studies, OCBs in NMOSD patients are present in 15–30% of cases [85,86], sometimes transiently detectable at the time of an attack [86]. During acute attacks of NMOSD pleocytosis is common, present in around 50% of samples and is useful in distinguishing NMOSD from MS. Typically cell analysis demonstrates neutrophils, eosinophils and activated lymphocytes [61].

### 4.6. Treatment

#### 4.6.1. Acute Treatment

NMOSD therapy includes the management of acute attacks and preventive treatment. All patients with suspected NMOSD should be treated in the acute phase because relapses can cause permanent disability [87]. Given the absence of controlled studies, in children treatment should performed based on the experiences in adults. A common approach to therapy begins with high dose intravenous methylprednisolone (IVMP). The recommended daily dose is 20 mg/kg/day to a maximum of 1000 mg for 5 consecutive days, followed by a gradual decrease to oral glucocorticoid and long-term maintenance [88]. If the initial response to steroids is insufficient, then PLEX should be considered [89]. Treatment with PLEX consists of 5 exchanges over 5–10 days [90]. In their study, Abboud et al. found that PLEX + IVMP was associated with improved outcomes compared to IVMP alone, especially in patients taking preventive treatment [91]. If PLEX is not possible for contraindications, IVIG (a total dose of 2 g/kg) is recommended [56]. However, the efficacy of IVIG is uncertain; indeed, only a few studies have evaluated the effect of IVIG on acute exacerbation of NMOSD [92,93]. In the acute phase all patients in our cohort underwent treatment with IVMP, while only two also required therapy with PLEX.

There are no evidence-based guidelines for the acute treatment of children with MOG-IgG. Usually, it consists of IVMP (20 mg/kg/day, maximum 1 g for 3–5 days), IVIG (total of 2 g/kg) and PLEX administered individually or in combination, depending on age and clinical presentation [94].

#### 4.6.2. Preventive Treatment

Preventive treatment is usually started after the first attack due to the risk of severe disability associated with each relapse. The rationale for the use of immunosuppressive therapy in pediatric patients is based on the experience in adults, in which preventive agents reduce the frequency and severity of relapses [18,95]. Practice generally recommends at least 5 years of maintenance therapy [96]. The current therapy options in pediatric patients include the off-label use of IVIG, AZA, MMF and RTX.

##### Intravenous Immunoglobulin

IVIG has a potential role in treatment of NMOSD both for therapy of acute attacks and as preventive therapy [97]. It is an efficacious therapy in other antibody-mediated neurological diseases [57]. It is still unknown the exact anti-inflammatory mechanism of action of IVIG. Probably, IVIG acts on the immune system through various mechanisms, such as neutralization of autoantibodies, binding of the antibody target, inhibition of dendritic cell activation and leukocyte migration, complement inhibition, and blocking of Fcγ receptors [98].

There are few studies in favor of the use of IVIG as preventive therapy in NMOSD. In a small study six NMO/NMOSD patients treated with 2 to 3 monthly IVIG infusions experienced a reduced number of relapses [98]. Another study reported successful reduction in relapse rates and good safety and tolerance in eight NMOSD patients treated with IVIG as preventive therapy [99]. In our series, one patient with seronegative NMOSD (case 7) is undergoing chronic therapy with cycles of IVIG, with good control of the disease.

##### Azathioprine

AZA is an immunosuppressive agent that acts through its effects as an antagonist of purine metabolism, resulting in the inhibition of DNA, RNA, and protein synthesis and consequently inhibition of cell proliferation, especially T and B cells [100]. A study of adult and pediatric AQP4-IgG NMOSD patients reported a modest efficacy of AZA [101]. Eighty-nine per cent of patients reported reduction in relapse rates and 61% remained relapse free at a median follow-up of 18 months with 2–3 mg/kg/day of AZA, but at last follow-up, treatment was discontinued in 46% due to adverse events. AZA treatment can have frequent side effects which may lead to intolerance. Common adverse events are nausea, vomiting, diarrhea, fever, thrombocytopenia, hepatotoxicity, leukopenia, and infections. One patient of ours with AQP4-IgG positive NMSOD (case 2) was treated with AZA with poor disease control.

##### Mycophenolate Mofetil

MMF is metabolized in the liver to the active mycophenolic acid. It is an inhibitor of inosine monophosphate dehydrogenase involved in guanosine nucleotide synthesis, used in the proliferation of B and T lymphocytes [102]. MMF is largely used in a variety of autoimmune diseases, included adult and pediatric NMOSD patients. In their observational study with NMOSD patients, Huh et al. reported that MMF therapy induced reduction of relapses, stabilized or improved disability, and was well tolerated [103]. In a retrospective study of 24 patients, 79% of NMOSD patients treated with MMF (median dose of 2000 mg per day) reported reduction in relapse rates and, in 91% of patients, reduction or stabilization of disability [104]. In pediatric age the recommended dose is 600 mg/m^2^/dose twice a day [105].

##### Rituximab

RTX is an anti-CD20 monoclonal antibody that causes B cell depletion by binding to the CD20 antigen of B cell lymphocytes [106].

Several studies on children and adults with NMOSD have demonstrated that RTX induces reduction in the annualized relapse rate, it is well-tolerated and stabilizes or improves neurologic disability [50,52,106,107].

The recommended starting dose in pediatric patients is 375 mg/m^2^ weekly for 4 consecutive weeks or 500 mg/m^2^/dose (max 1 g), two infusions, 2 weeks apart [107], with monitoring of CD19 cell counts [50].

Common side effects include rash, a flu-like syndrome, headache, nausea, and fatigue, especially with the first infusion [50]. To reduce these reactions, pretreatment with acetaminophen, diphenhydramine and corticosteroids is recommended [108].

Patients should be screened also for hypogammaglobulinemia before and after administration of RTX, due to the risk of prolonged hypogammaglobulinemia and associated infections [106,109]. We had two patients, one with positive NMOSD (case 1) and the other seronegative (case 4) treated with MMF associated with RTX that showed a good control of disease. One patient with positive NMOSD (case 3) had a good response to RTX with an improvement in neurological disability, while another seronegative patient (case 5) had a partial response to RXT. Only one patient with AQP4-IgG (case 2) had no disease control after treatment with RTX.

##### Preventive Treatment in MOG-IgG Positive Patients

In MOG-IgG positive patients, chronic maintenance therapy is reserved for those with relapsing disease [94]. In a large retrospective multicenter study of adult and pediatric patients with MOG-IgG associated disorder, IVIG therapy was associated with the greatest reduction in relapse rate, as compared to other maintenance therapies (RTX, AZA, MMF) [99]. In a multinational European cohort study of 102 children, AZA, MMF, RTX, and particularly IVIG were associated with a reduction in relapse frequency [110]. In this study as well, maintenance with IVIG was found superior to other therapies.

#### 4.6.3. New Therapeutic Agents

Several new therapeutic agents for NMOSD are being evaluated in trials in adult patients.

##### Eculizumab

Eculizumab is an anti-C5 monoclonal antibody. In a randomized double-blind placebo-controlled trial of 143 adult with AQP4-IgG-positive NMOSD, treatment with intravenous eculizumab reduced the risk of relapse [111]. Eculizumab is generally well tolerated. The most frequent side effects included upper respiratory tract infections, headache, diarrhea, and nausea [111]. The most severe adverse event is the increased risk of meningococcal and encapsulated bacterial infection [112]. For this reason, patients should be immunized with meningococcal vaccines. A trial that evaluates the safety and efficacy of eculizumab in pediatric patients with relapsing NMOSD is ongoing [113].

##### Tocilizumab

Tocilizumab is a monoclonal antibody against the IL-6 receptor. Based on different studies, it is a promising therapeutic option for NMOSD. Araki M. et al. reported that tocilizumab induced reduction of annualized relapse rate. Their study showed the efficacy and good safety of tocilizumab [114].

##### Satralizumab

As tocilizumab, satralizumab is a recombinant monoclonal antibody targeting interleukin-6 receptors [115]. In a phase three, randomized, double-blind, placebo-controlled trial with seronegative and seropositive NMOSD patients, satralizumab had a greater effect than placebo on reduction of the relapse rate, with a better response in AQP4-IgG seropositive patients [115]. There is an ongoing trial that evaluate the safety and efficacy of satralizumab in pediatric participants with NMOSD [116].

##### Inebilizumab

Inebilizumab is an anti-CD19 monoclonal antibody. Compared with anti-CD20 antibodies that deplete a small subset of B lymphocytes, anti-CD19 antibodies deplete a wider range of lymphocytes derived from the B-cell lineage [117]. In a trial with 230 seronegative and seropositive NMOSD patients, inebilizumab reduced the risk of relapse, disability, and MRI activity [118].

##### Hematopoietic Stem Cell Transplantation

The treatment of refractory NMOSD remains a considerable challenge for neurologists. In these NMOSD patients, Hematopoietic Stem Cell Transplantation (HSCT), both autologous (au HSCT) and allogeneic (al HSCT) HSCT should be considered as a possible therapeutic option in the most severe form of NMOSD [119]. Ceglie et al. reviewed the HSCT applications in NMOSD patients. Although the experience is limited, they observed that al HSCT is superior in maintaining long-term stabilization of the disease compared to au HSCT [55]. In our series, case 2 was treated with an HLA-haploidentical HSCT that led to clinical and radiological stability. This is the first case of a pediatric patient to benefit from such a treatment.

**Figure 3:**
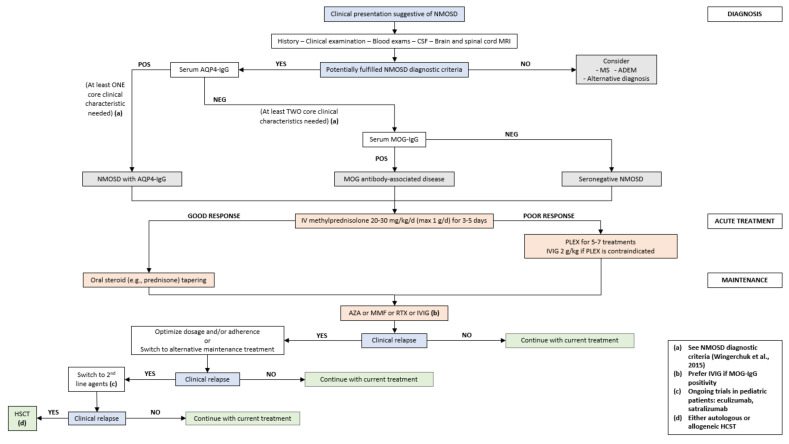
Proposed diagnostic and treatment algorithm for pediatric NMOSD. NMOSD: neuromyelitis optica spectrum disorders; CSF: cerebrospinal fluid; MRI: magnetic resonance imaging; MS: multiple sclerosis; ADEM: acute disseminated encephalomyelitis; AQP4: aquaporin-4; MOG: myelin oligodendrocyte glycoprotein; IV: intravenous; PLEX: plasma exchange; IVIG: intravenous immunoglobulin; AZA: azathioprine; MMF: mycophenolate mofetil; RTX: rituximab; HCST: hematopoietic stem cell transplantation.

Proposed diagnostic and treatment algorithm for pediatric NMOSD is shown in Figure 3.

## 5. Conclusions

Here, we have reviewed recent literature to provide a tool for diagnosing and treating children with NMOSD. These case reports are an example of the diagnostic and therapeutic complexity of pediatric NMOSD. Our cases, although limited in number, offer a wide range of personalized therapeutic strategies for the individual patient. Furthermore, our series also discusses AQP4-negative patients, unlike the other reviews that focus on AQP4-positive pediatric patients.

In pediatric patients, diagnosis of NMOSD is often difficult, especially for patients who are seronegative for MOG and AQP4 and consequently the choice of therapy. Treatment options for pediatric patients with NMOSD are available and will continue to expand.

Thanks to new discoveries on the neurophysiological mechanisms underlying NMOSD, many new pharmacological agents are being studied, especially in the adult population.

However, further studies on long-term safety and treatment response of newer therapeutic agents are necessary, especially with regard to seronegative patients.

## Figures and Tables

**Table 1:** Clinical, radiological and laboratory findings of our patients with NMOSD.

Pt	Gen, Age (y)	Symptoms/Signs	MRI T2-Hyperintense Lesions	Gd Enhancement	Antibody Status	CSF OCBs	Evoked Potentials	EDSS at the Onset	Acute Attack Therapy	Clinical Course	Long-Term Therapy	EDSS at Last Follow-Up
1	F, 16	Headache, vomiting, vertigo	Right centrum semiovale, near the trigone and the temporal horn of the right ventricle, pons, medulla oblongata, dorsal spine	Yes	AQP4-IgG positive	No	Normal VEPs, SEPs and MEPs	3	IVMP	Monophasic	Oral CS, HCQ, MMF	0
2	F, 9	Bilateral ON, progressive bilateral lower limb hyposthenia	Right frontal subcortical region, corpus callosum, cervical and dorsal spine	Yes	AQP4-IgG positive	Yes	Abnormal VEPs and SEPs (MEPs not available)	4	IVMP	Relapsing	Oral CS, RTX, AZA	5
3	F, 8	Bilateral ON	Bilateral optic nerves	Yes, left optic nerve	AQP4-IgG positive	No	Abnormal VEPs, normal SEPs and MEPs	4	IVMP, PE	Monophasic	RTX	0
4	M, 15	Progressive right lower limb paresis	From cervical spine to the conus medullaris	No	AQP4-IgG and MOG-IgG negative	No	Abnormal VEPs, SEPs and MEPs	8.5	IVMP	Relapsing	RTX, MMF	0
5	M, 10	Right lower limb paresis, sensory level at T10-T11	Diffuse bilateral cerebral involvement, cervical spine	Yes	AQP4-IgG and MOG-IgG negative	No	Abnormal VEPs, SEPs and MEPs	9	IVMP, PE	Relapsing	RTX	7.5
6	M, 13	Gait ataxia, bilateral lower limb paresthesia, pyramidal signs, sensory level at T10	Dorsal spine	Yes	AQP4-IgG and MOG-IgG negative	No	Abnormal VEPs (SEPs and MEPs not available)	6	IVMP	Monophasic	Oral CS	0
7	M, 11	Unilateral ON	Left optic nerve and cervical spine	Yes	AQP4-IgG and MOG-IgG negative	No	Abnormal VEPs, normal SEPs and MEPs	2	IVMP	Relapsing	IVIG	1

Pt: patient; y: years; Gen: gender; CSF: cerebrospinal fluid; OCBs: oligoclonal bands; Gd: gadolinium; EDSS: Expanded Disability Status Scale; ON: optic neuritis; PE: plasma exchange; IVMP: intravenous methylprednisolone; CS: corticosteroids; MMF: mycophenolate mofetil; RTX: rituximab; HCQ: hydroxychloroquine; AZA: azathioprine; IVIG: intravenous immunoglobulin.

**Table 2:** Main characteristics of all studies selected for this review.

Reference	Study Type	N	Age Range (years)	Diagnosis	AQP4 +	MOG +	Seronegative NMOSD	Diagnostic Criteria	Symptoms at Onset	Treatment	Outcome
Fragoso YD et al., 2014 [16]	Case series	n = 29	13 ± 3.4 (5–17)	NMO	n = 22	NA	Negative (n = 5), not performed (n = 2)	Wingerchuk et al., 2006	Myelitis (n = 12), ON (n = 8), myelitis and ON (n = 9)	AZA (n = 29), AZA + PDN (n = 4), AZA + GA (n = 3), IVIG (n = 6), PLEX (n = 2), MTX + PDN (n = 1)	NA
Paolilo RB et al., 2020 [17]	Retrospective study	n = 67	10.2 ± 3.6	NMOSD	n = 67	NA	NA	Wingerchuk et al., 2015	ON (n = 20), TM (n = 15), area postrema syndrome (n = 11), simultaneous ON and TM (n = 6), ADEM (n = 6), isolated diencephalic syndrome (n = 1), isolated brainstem syndrome (n = 3), multifocal syndromes (n = 5)	1 DMT (n = 41), 2 DMTs (n = 12), 3 DMTs (n = 7), 4 DMTs (n = 2), 5 DMTs (n = 1), untreated (n = 4)	EDSS ≥ 3 (n = 29), visual impairment (n = 32), motor deficits (n = 14), cognitive impairment (n = 17)
Absoud M et al., 2015 [8]	Retrospective study	n = 20	10.5 (2.9–16.8)	NMO	n = 8	NA	n = 12	Wingerchuk et al., 2006	ON (n = 12), TM (n = 3), ON + TM (n = 3), ADEM (n = 2)	Oral PDN, AZA, RTX, IVIG, MMF, mitoxantrone, ofatumumab	Visual impairment (n = 10), wheelchair-dependent (n = 3)
Chitnis T et al., 2016 [18]	Prospective multicenter database	n = 38	10.2 ± 4.7	NMO NMOSD	n = 24	NA	n = 14	Wingerchuk et al., 2006 Wingerchuk et al., 2015	Visual (n = 21), motor (n = 17), constitutional (n = 23) symptoms	RTX (47%), MMF (39%), AZA (24%), PLEX (39%)	Number of attacks in first two years (1.84 ± 1.44)
Khan TR et al., 2020 [19]	Case report	n = 1	2	NMOSD	n = 1	NA	n = 0	Wingerchuk et al., 2015	TM (progressive left-hand weakness, gait instability)	IVMP, PLEX, RTX, MMF	Several exacerbations
Gmuca S et al., 2017 [20]	Case series	n = 4	11–15	NMOSD + SS	n = 4	NA	n = 0	Wingerchuk et al., 2015	TM + ON (n = 1), ON (n = 2), intractable emesis and hiccups (n = 1)	IVMP, RTX, CP, PLEX, HCQ, MMF	NA
Maraş H et al., 2013 [21]	Case report	n = 1	5.67	NMO	n = 1	NA	n = 0	Krupp et al., 2007	ON	IVMP, oral CS, AZA	Several relapses with vision loss
Mahmood NA et al., 2011 [22]	Case series	n = 2	10–15	NMO	n = 2	NA	n = 0	Wingerchuk et al., 2006	TM (n = 2)	IVMP, oral CS, IVIG, AZA, RTX	Relapses (ON, bowel and bladder retention, leg weakness)
Ikeda A et al., 2019 [23]	Retrospective study	n = 4	8 (3–12)	NMOSD	n = 0	n = 4	n = 0	Wingerchuk et al., 2015	ON (n = 2), extremity weakness/paresthesia (n = 2)	IVMP, oral CS, PLEX, AZA, tacrolimus	Several relapses, but no sequelae
Chuquilin M et al., 2016 [24]	Case report	n = 2	3–3.5	NMO	n = 1	NA	NA	Wingerchuk et al., 2015	Blindness (n = 2)	GA, AZA, CS, IFN-beta1a, RTX, PLEX	Several relapses
Lotze TE et al., 2018 [25]	Retrospective study	n = 9	14 (1.9–16)	NMOSD	n = 7	NA	NA	Wingerchuk et al., 2006	Visual, motor, and mixed symptoms	IVMP, IVIG, PLEX, CP, MMF, RTX, AZA, GA	Relapsing-remitting course in all patients
Rostásy K et al., 2013 [26]	Retrospective study	n = 8	10.5 (3–15)	NMO	n = 2	n = 3	n = 3	Wingerchuk et al., 2006	Various	RTX, AZA, oral CS, IVIG	Monophasic (n = 1), relapses (n = 7)
Dimitrijevic N et al., 2012 [27]	Case report	n = 1	3	NMO	n = 1	NA	NA	Wingerchuk et al., 2006	Isolated ON (sudden visual loss)	IVMP, oral CS, IVIG	Several relapses leading to blindness, paraplegia, urinary and bowel incontinence, short stature
Loma IP et al., 2008 [28]	Case report	n = 1	3.9	NMO	n = 1	NA	n = 0	Wingerchuk et al., 2006	Bilateral leg weakness, urinary and bowel incontinence (LETM)	IVMP, oral CS, IVIG, AZA	Several relapses, but no sequelae
Yavuz H et al., 2013 [29]	Case report	n = 1	13	NMO	NA	NA	NA	Sellner et al., 2010	Visual loss, behavioral changes	CS, IVIG, PLEX, AZA	Several relapses with vision impairment
Marino A et al., 2017 [30]	Case report	n = 1	1.9	NMO + SS	n = 1	NA	n = 0	Wingerchuk et al., 2015	Central pontine myelinolysis, unilateral ON, ON + TM	IVIG, IVMP, PLEX, HCQ, CP, RTX, MMF, TCZ	Several relapses before TCZ introduction
He D et al., 2014 [31]	Case report	n = 1	5	NMO	n = 1	NA	n = 0	Krupp et al., 2007	Bilateral ON	IVMP, oral CS, IVIG, RTX	Several relapses before RTX introduction
Elpers C et al., 2015 [32]	Case report	n = 1	12	NMO	n = 1	NA	n = 0	Wingerchuk et al., 2006	Left arm paresis, left leg progressive paresis, dizziness, neck pain (LETM)	IVMP, oral CS, PLEX, AZA, RTX	Relapses before RTX introduction
Maillart E et al., 2020 [33]	Case report	n = 1	8	NMOSD	n = 1	NA	n = 0	Wingerchuk et al., 2015	Bilateral ON + LETM	IVMP, oral CS, PLEX, MMF, RTX, ofatumumab	Several relapses with permanent visual disability
Kamawal A et al., 2019 [34]	Case report	n = 1	6	NMOSD	n = 0	n = 1	n = 0	Wingerchuk et al., 2015	Progressive headache, meningism	IVMP, PLEX, MMF	Neurological restitutio ad integrum within two months
Hudson LA et al., 2006 [35]	Case report	n = 1	8	NMO	n = 1	NA	n = 0	Wingerchuk et al., 2006	Bilateral upper extremity paresthesia	IVMP, oral CS	One relapse
Gokce G et al., 2013 [36]	Case report	n = 1	10	NMO	n = 1	NA	n = 0	Wingerchuk et al., 2006	Sudden visual loss	IVMP, oral CS, AZA	Significant bilateral optic atrophy
Khan TR et al., 2021 [37]	Case report	n = 1	2	NMOSD	n = 1	NA	n = 0	Wingerchuk et al., 2015	Left-hand weakness and abnormal gait (LETM)	PLEX, RTX, MMF, TCZ, autologous HSCT	Highly active disease with several hospitalizations
Arnold TW et al., 1987 [38]	Case report	n = 1	12	NMO	NA	NA	NA	NA	Decreased vision in the right eye, paraparesis, bilateral leg pain	PDN	Normal neurologic examination eight months after discharge
Davis R et al., 1996 [39]	Case report	n = 1	4	NMO	NA	NA	NA	NA	TM	IV and oral dexamethasone	One relapse (ON); unremarkable neurologic examination after 18 months
Peña JA et al., 2011 [40]	Cohort study	n = 6	11 (5–13)	NMO	n = 4	NA	Negative (n = 1), not performed (n = 1)	Wingerchuk et al., 2006	ON (n = 3), TM (n = 1), ON + TM (n = 2)	IVMP, IVIG, PDN, AZA, IFN-beta1a	Relapsing-remitting course with bilateral vision loss and paraparesis in all patients
Numata Y et al., 2015 [41]	Case report	n = 1	9	NMO	n = 1	NA	n = 0	Wingerchuk et al., 2006	Nausea, vomiting, intractable hiccups	IVMP, IVIG, oral PDN	Recurrence-free for 10 months after discharge
Milani N. et al., 2004 [42]	Case report	n = 1	7	NMO	NA	NA	NA	Wingerchuk et al., 1999	Fever and vomiting, followed by headache, neck stiffness and visual impairment	IVMP, IVIG, PDN, CP, AZA	Relapsing-remitting course: paraparesis with normal brain MRI
Longoni G et al., 2014 [43]	Case report	n = 1	10	NMOSD	n = 1	NA	n = 0	NA	Subacute lower limb weakness, gait ataxia	Targeted immunotherapy	Partial lesion resolution
Bianchi A et al., 2017 [44]	Case report	n = 1	7	NMOSD	n = 1	NA	n = 0	Wingerchuk et al., 2015	Cervical back pain, paraparesis	IVMP, oral CS, AZA, RTX	Three episodes of isolated recurrent myelitis with no further relapses after RTX introduction
Arabshahi B et al., 2006 [45]	Case report	n = 1	11	NMO + SS	NA	NA	NA	NA	Bilateral ON	CS, PLEX, CP	Visual impairment
Zhang Z et al., 2021 [46]	Case series	n = 11	7 (5–13)	NMOSD	n = 2 (n = 1: both AQP4 and MOG)	n = 2 (n = 1: both AQP4 and MOG)	Negative (n = 5), not performed (n = 3)	Wingerchuk et al., 2015	ON (n = 4), ON + encephalopathy (n = 5), myelitis + encephalopathy (n = 2)	NA	NA
Baghbanian SM et al., 2019 [47]	Case series	n = 10	13 (8–17)	NMOSD	n = 7	NA	NA	Wingerchuk et al., 2015	ON (n = 4), ON + TM (n = 1), LETM (n = 3), APS (n = 2)	IVMP, PLEX (n = 5), AZA, RTX (n = 5)	Favorable: EDSS before preventive therapy was 3 (range 0–5) and decreased to 2.5 (range 0–5) after preventive therapy
Dembinski K et al., 2013 [48]	Case report	n = 1	13	NMO	n = 1	NA	n = 0	Wingerchuk et al., 2006	Encephalopathy with waxing and waning mental status, flat affect, ophthalmoparesis, with decreased visual acuity and color agnosia	IVIG, IVMP, RTX	Favorable
Farhat L et al., 2018 [49]	Case report	n = 1	17	NMO	NA	NA	NA	NA	Acute vision loss in the left eye	AZA, CS, RTX, IGRT	Secondary hypogammaglobulinemia following RTX administration
Nosadini M et al., 2016 [50]	Multicenter retrospective study	n = 16	1.8–15.3	NMO NMOSD	NA	NA	NA	Wingerchuk et al., 2015	ON (n = 4), TM (n = 4), ON+TM (n = 2), BD (n = 3), TM + BD (n = 2), ON + BD (n = 1)	Before RTX, 62.5% had received AZT, MMF, or CP. Then all 16 patients had ≥ 2 RTX courses	6 patients were relapse-free, although 21 relapses occurred in 10 patients
Lechner C et al., 2020 [51]	Multicenter pro- and retrospective study	n = 24	11 (3–17)	NMOSD	n = 16	n = 4	n = 3	Wingerchuk et al., 2015	LETM (n = 5), BS (n = 3), bilateral ON (n = 1), APS (n = 1), LETM + bilateral ON (n = 4), LETM + BS (n = 2), LETM + unilateral ON (n = 1), LETM + BS + bilateral ON (n = 1)	Acute: IVM, IVIG (n = 7), PLEX (n = 7), RTX (n = 2). Long-term: RTX (n = 8), AZA (n = 4), TCZ (n = 2), MMF (n = 1), IVIG (n = 1), CP (n = 1)	NA
Longoni G et al., 2014 [52]	Retrospective observational cohort study	n = 5	10.9–17	NMO NMOSD	n = 5	NA	n = 0	NA	ON (n = 1), hiccups with nausea and vomiting (n = 2), gait disturbances (n = 1), hiccups with nausea and vomiting and progressive encephalopathy (n = 1)	Acute: IVMP, IVIG (n = 3), RTX (at 6 months and 12 months)	Favorable: EDSS score in the 5 patients decreased from 3.0 at initiation of RTX to 2.0 at 6 months from onset and 0.8 at 12 months from onset
Camera V et al., 2021 [53]	Retrospective multicenter cohort study	n = 49	12 ± 4.1	NMOSD	n = 49	NA	n = 0	NA	Multifocal onset presentation (26.5%), optic nerve (47%), area postrema/brainstem (48.9%), encephalon (28.6%)	NA	NA
Bradshaw MJ et al., 2017 [54]	Case report	n = 1	3	NMO	n = 1	NA	n = 0	Wingerchuk et al., 2015	Progressive bilateral vision loss, left Babinski sign	5 sessions of PLEX/IVMP, RTX	Favorable: vision improvement after IVMP and after first cycle of RTX
Ceglie G et al., 2019 [55]	Case report	n = 1	9	NMO	n = 1	NA	n = 0	NA	Bilateral ON and progressive hyposthenia at the lower limbs	High-dose CS, AZA, cyclosporine, RTX, allogeneic HSCT	Favorable: no re-exacerbation, with long-term stabilization

NMO: neuromyelitis optica; NMOSD: neuromyelitis optica spectrum disorder; SS: Sjogren’s syndrome; MOG: myelin oligodendrocyte glycoprotein; AQP4: aquaporin-4; +: positive; ON: optic neuritis; TM: transverse myelitis; ADEM: acute disseminated encephalomyelitis; LETM: longitudinally extensive transverse myelitis; APS: area postrema syndrome; BD: brainstem disease; BS: brainstem syndrome; IVMP: intravenous methylprednisolone; CS: corticosteroid; AZA: azathioprine; PDN: prednisone; GA: glatiramer acetate; IVIG: intravenous immunoglobulin; PLEX: plasma exchange; MTX: methotrexate; RTX: rituximab; DMT: disease modifying therapy; MMF: mycophenolate mofetil; CP: cyclophosphamide; HCQ: hydroxychloroquine; IFN-beta1a: interferon-beta1a; TCZ: tocilizumab; IGRT: immunoglobulin replacement therapy; HSCT: hematopoietic stem cell transplantation; EDSS: Expanded Disability Status Scale; MRI: magnetic resonance imaging; NA: not available.
